# Circulating Endothelial Progenitor Cells in Type 1 Diabetic Patients: Relation with Patients’ Age and Disease Duration

**DOI:** 10.3389/fendo.2017.00278

**Published:** 2017-10-23

**Authors:** Adolfo Arcangeli, Elena Lastraioli, Barbara Piccini, Massimo D’Amico, Lorenzo Lenzi, Serena Pillozzi, Maria Calabrese, Sonia Toni, Annarosa Arcangeli

**Affiliations:** ^1^Diabetology Unit, Prato Hospital, Prato, Italy; ^2^Department of Experimental and Clinical Medicine, University of Florence, Florence, Italy; ^3^Diabetology Unit, Azienda Ospedaliero Universitaria Meyer, Florence, Italy; ^4^DI.V.A.L Toscana Srl, Sesto Fiorentino, Italy

**Keywords:** type 1 diabetes mellitus, endothelial progenitor cells, flow cytometry, diabetes duration, patients’ age

## Abstract

**Objectives:**

Circulating endothelial progenitor cells (cEPCs) have been reported to be dysfunctional in diabetes mellitus (DM) patients, accounting for the vascular damage and the ensuing high risk for cardiovascular disease (CVD) characteristic of this disease. The aim of the present study was to evaluate the number of circulating cEPCs in type 1 DM (T1DM) patients, without clinical vascular damage, of different ages and with different disease duration.

**Methods:**

An observational, clinical-based prospective study was performed on T1DM patients enrolled in two clinical centers. cEPCs were determined by flow cytometry, determining the number of CD34/CD133/VEGFR2-positive cells within peripheral blood mononuclear cells (PBMCs).

**Results:**

The number of cEPCs was lower in adult T1DM patients, whilst higher in childhood/young patients, compared to controls of the same age range. When patients were grouped into two age groups (≥ or <20 years) (and categorized on the basis of the duration of the disease), the number of cEPCs in young (<20 years) patients was higher compared with older subjects, regardless of disease duration. A subset of patients with very high cEPCs was identified in the <20 years group.

**Conclusion:**

There is an association between the number of cEPCs and patients’ age: childhood/young T1DM patients have significantly higher levels of cEPCs, respect to adult T1DM patients. Such difference is maintained also when the disease lasts for more than 10 years. The very high levels of cEPCs, identified in a subset of childhood/young patients, might protect vessels against endothelial dysfunction and damage. Such protection would be less operative in older subjects, endowed with lower cEPC numbers, in which complications are known to develop more easily.

## Introduction

Diabetes mellitus (DM) is characterized by long-term vascular damage to small vessels and major arteries and by an impaired vascular repair, which collectively leads to a higher risk of cardiovascular disease (CVD) (1). Contributory factors to the vascular impairment in DM include increased glucose level, other traditional cardiovascular risk factors, arterial wall inflammation, and endothelial dysfunction (2). Endothelial dysfunction is considered the pivotal mechanism sustaining vascular injury, and hence the heightened cardiovascular burden in DM (3).

After a vascular injury, endothelial repair depends both on the migration and proliferation of endothelial cells of the vascular wall and by the arrival of endothelial progenitor cells (EPCs) from the bone marrow in the site of damage (4, 5). The release of EPCs from the bone marrow depends on the stimulatory effect of different growth factors/cytokines, such as VEGF and IL-8 (6, 7). In this light, a novel paradigm of CVD pathogenesis is the loss of normal endothelial turnover caused by a reduction of EPCs [reviewed by Shantsila et al. (8)].

A reduction of circulating EPCs (cEPCs) has been hypothesized to promote the development and/or progression of vascular dysfunction and CVD in DM (9). EPC dysfunction would also represent the molecular transducer in the mechanism through which risk factors negatively affect cardiovascular function in DM (10). Consistently, several reports have shown that both the number and functionality of cEPCs are reduced in type 2 DM (T2DM) and that such impairment is related to the morphological and functional alterations detected in peripheral vessels (9, 11–13). Furthermore, T2DM patients show low serum levels of those growth factors/cytokines known to trigger EPC release from the bone marrow, accounting for a reduced bone marrow stimulation and hence EPCs release (14). The levels of cEPCs and arterial wall stiffness in T2DM subjects are strictly correlated with glycemic control (15, 16). Interestingly, a clear correlation between EPC activity, glycemic control, and myocardial savage has been recently demonstrated (17). Hyperglycemia may *per se* affect EPC number and functional capacity, because it enhances EPC senescence and triggers apoptosis (18). Sirtuins have been identified as molecular mediators of the deleterious effect of hyperglycemia in EPCs (16, 19).

The scenario is apparently similar in Type 1 DM (T1DM), where a general reduction of cEPC number has been reported (20–24). Only Głowińska-Olszewska et al. (25) showed that contrary to adult population with diabetes, T1DM diabetic children have an increased number of EPCs.

Based on the latter data and on the clinical observation of less incidence of late vascular complications in T1DM when the onset of the disease is in childhood respect to adult age (26), we undertook a study aimed at determining the number of cEPCs in T1DM patients without clinical vascular damage, of different ages and disease duration.

## Materials and Methods

### Study Population

We performed an observational, clinical-based prospective study on type 1 diabetic patients treated at two different Italian centers: the Diabetic Unit of the Meyer Hospital in Florence and the Diabetic Unit of the Prato Hospital, Prato. The Meyer Hospital specifically enrolled childhood (age <10 years) and young (age 10–24 years) T1DM patients and age-matched controls; the Diabetic Unit of the Prato Hospital enrolled adult (25–59 years) T1DM patients and age-matched controls. Patients were enrolled after informed written consent in accordance with the Declaration of Helsinki. The study was approved by Local Ethical Committee. The enrollment started in January 2010 and ended on May 2012; patients were followed up until December 2014. Inclusion criteria were the clinical diagnosis of T1DM from at least two years and the lack of clinical CV complications. In particular, patients were screened for hypertension, coronary artery disease, peripheral arterial disease, peripheral neuropathy, retinopathy, and nephropathy.

Twenty-two healthy individuals, selected within the same age range as T1DM patients, were enrolled as controls. None of the controls had a clinical history of diabetes. They had normal fasting blood glucose levels and normal physical examination and had not received any medication.

### Sample Preparation

Mononuclear cells from peripheral blood mononuclear cell (PBMC) samples were isolated by density gradient centrifugation using Lympholyte (Cedarlane Laboratories, Burlington, ON, Canada). Briefly, 5 ml of peripheral blood were diluted 1:2 with PBS and the diluted blood was stratified onto 5 ml of Lympholyte. Samples were centrifuged 30 min at room temperature at 3,000 rpm without brake. After separation, white blood cells were recollected, diluted with PBS and centrifuged at 1,200 rpm for 5 min at room temperature. Subsequently, pellet was treated with Red Cell Lysis Buffer, to ensure red blood cell removal and washed in PBS.

### Fluorescence-Activated Cell Sorting (FACS) Analysis

**Figure 1:**
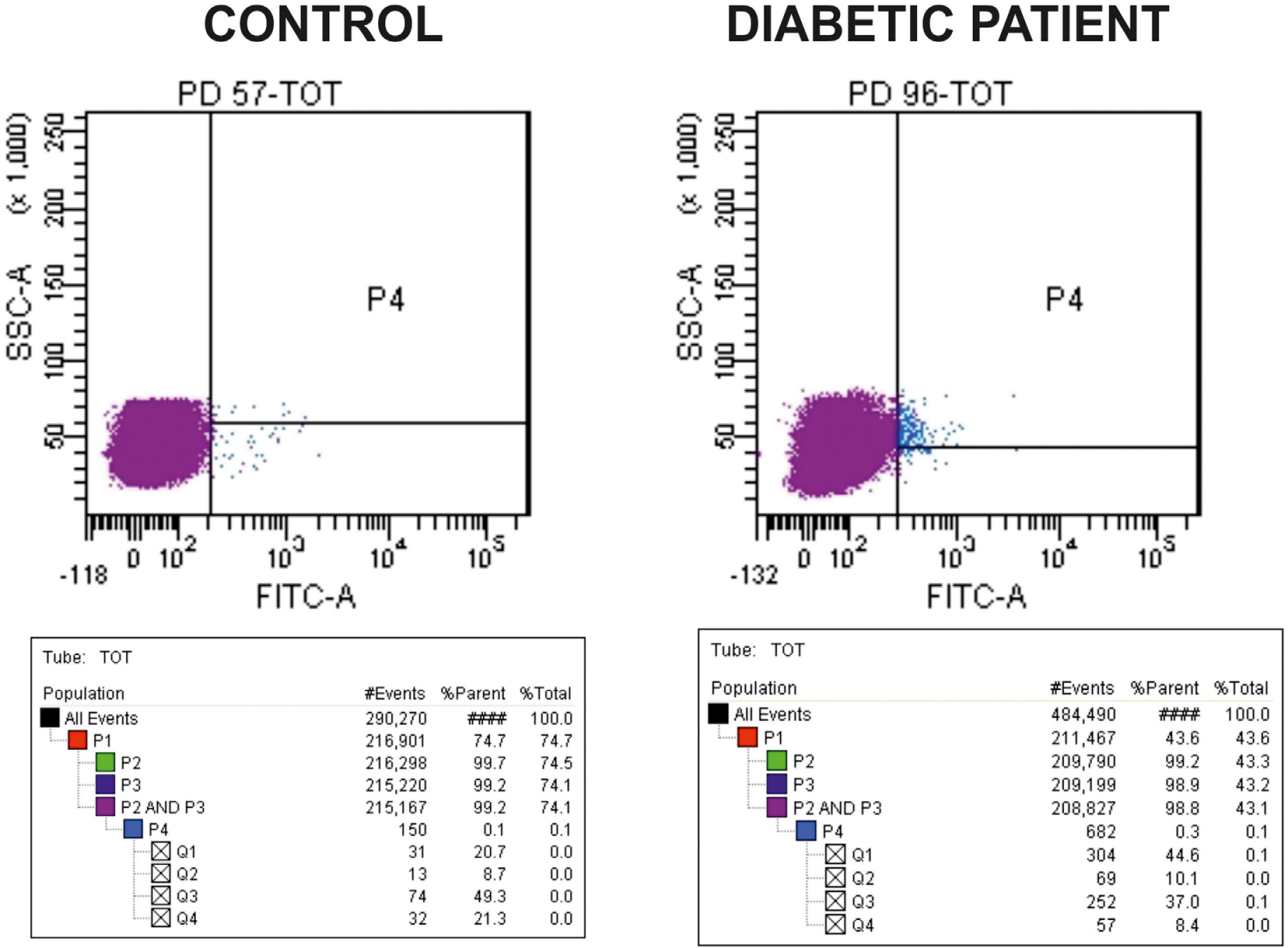
Representative dot-plots of a control subject [left panel, circulating endothelial progenitor cells (cEPCs) = 8.7] and diabetic patient belonging to the “<20 y T1DM” cohort (right panel, cEPCs = 10.1). The cEPC count was performed as described in the Section “Materials and Methods” and gating a population with morphological characteristics between lymphocytes and monocytes (evaluated through side scatter and forward scatter). In the final plots, the cEPC count of each sample is reported in Q2 that is a part of P4 quadrant. “cEPC counts” refers to the absolute cEPC number per 2 × 10^5^.

The number of cEPCs was assessed by flow cytometry by determining the number of CD34/CD133/VEGFR2-positive cells. In particular, 100 µl of each sample prepared as described in the previous section, were stained with 1 µl each of FITC-CD34 (BD Biosciences, Franklin Lakes, NJ, USA), APC-CD133/2 (Miltenyi Biotec, Bergisch Gladbach, Germany), and PE-VEGFR-2 (R&D Systems, Minneapolis, MN, USA), and incubated in the dark in ice for 15 min. For each sample, a control tube with no antibodies was prepared together with the stained tube. After washing the cells with PBS, FACS analysis was performed on a FacsCanto (BD Biosciences, Franklin Lakes, NJ, USA). The acquisition goal was 2 × 10^5^ events. Samples were analyzed gating a population with morphological characteristics between lymphocytes and monocytes, evaluated on the basis of side scatter and forward scatter parameters. As evident from Figure 1, where representative dot-plots relative to a control and a T1DM patient (belonging to the “<20 y T1DM cohort”) are shown, the cEPC count is reported in Q2, that is a part of the P4 quadrant. Throughout the manuscript, “cEPC counts” refers to the absolute cEPC number per 2 × 10^5^ PBMC.

### Statistical Analysis

Data are given as mean ± SEM. First of all, normality of the distribution of cEPC counts was assessed by Kolmogorov–Smirnov test. In order to apply the correct type of *T* test (for unpaired samples with equal or different variance) for normally distributed samples, the analysis of the variance was assessed by ANOVA test at the 0.05 level. Once determined the normality as well as the variance of the samples, the proper test was applied to evaluate differences among groups. In particular, we used the two-sided Student’s *t*-test for unpaired samples (either with different variance or with equal variance) for normally distributed data and the Mann–Whitney *U* test when samples were not normally distributed. In both cases, *p* < 0.05 was considered as significant. The Pearson correlation coefficient was calculated to evaluate relationships between cEPC count and clinical parameters.

### Study Design

**Figure 2:**
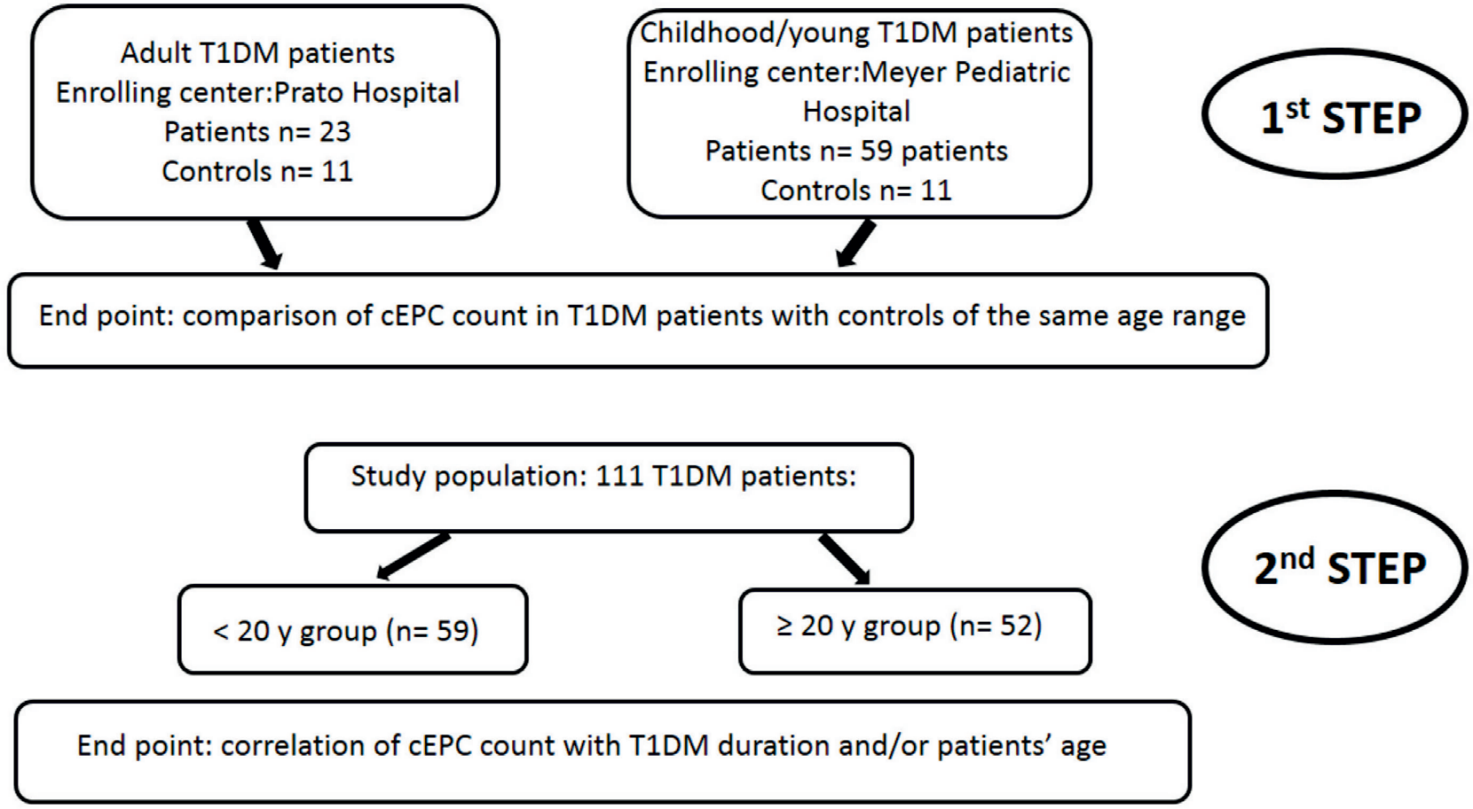
Flow diagram showing the rationale of the study. The end point of the first step of the study was the evaluation of the number of circulating endothelial progenitor cells (cEPCs) in adult and childhood/young patients, compared to age-matched controls. In the second step of the study, the end point was the association of the number of cEPCs with disease duration and/or patients’ age.

As depicted in the flow diagram shown in Figure 2, the study was divided into two steps. In the first step, two cohorts of T1DM patients enrolled in the two (adult and pediatric) diabetic centers were analyzed independently. The end point of the first step of the study was the evaluation of the number of cEPCs in adult and childhood/young patients, compared to controls of the same age range. In the second step, the study population (a total of 111 patients enrolled in both centers) was divided in two groups of similar numerosity, depending on the age (≥ or <20 years). The end point of this second step was the analysis of the association between the number of cEPCs and disease duration and/or patients’ age.

## Results

**Figure 3:**
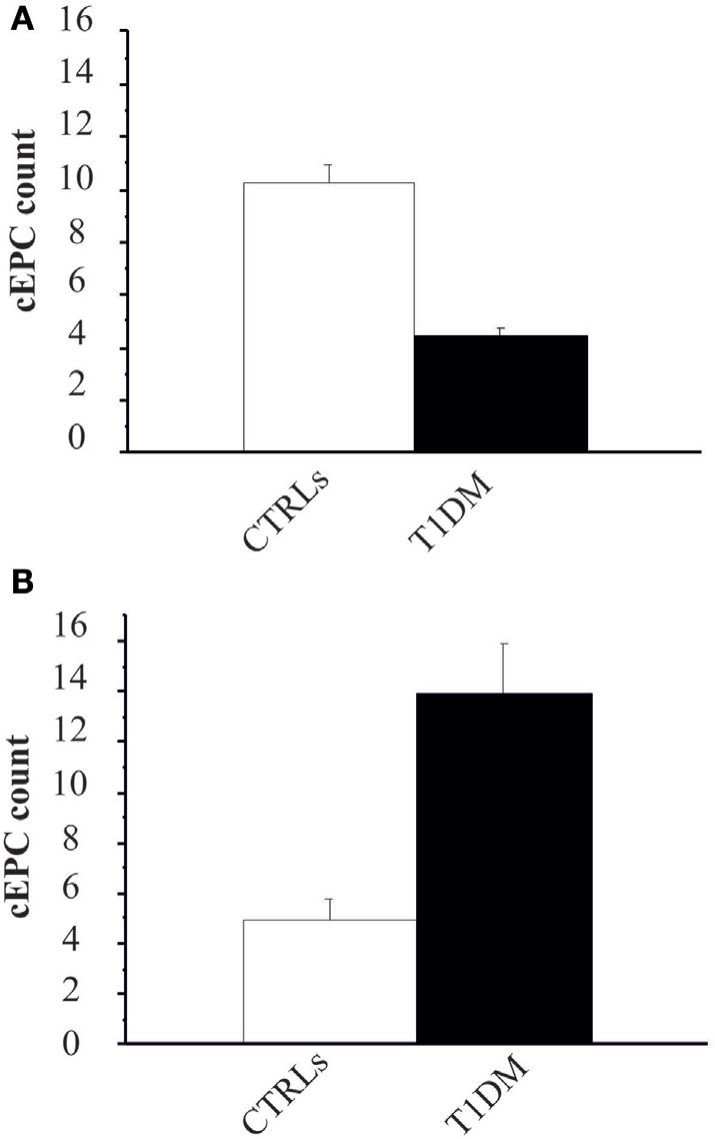
**(A)** Circulating endothelial progenitor cell (cEPC) counts (absolute cEPC number per 2 × 10^5^ peripheral blood mononuclear cell) in adult control subjects (white bar) and adult type 1 diabetes mellitus (T1DM) (black bar). Data are reported as mean ± SEM; *p* < 0.001, Student’s *t*-test. **(B)** cEPC count in childhood/young control subjects (white bar) and age-matched T1DM (black bar). Data are reported as mean ± SEM; *p* < 0.001, Mann–Whitney *U* test.

The first objective of our study was to determine the number of cEPCs in T1DM patients compared to healthy controls (see the flow diagram of the study in Figure 2). Two different patients’ cohorts were examined: one relative to adult T1DM patients enrolled in the Prato Hospital and one relative to childhood/young patients, enrolled in the Meyer Pediatric Florence Hospital. The clinical characteristics of T1DM patients enrolled in the two centers are shown in Tables 1 and 2, along with cEPC data. Note that for healthy controls, only gender and age data are reported in the tables. The number of cEPCs was significantly reduced in adult T1DM patients compared to controls within the same age range (4.43 ± 0.29 *n* = 23 vs 10.29 ± 0.68 *n* = 11; *p* < 0.001, Student’s *t*-test) (Figure 3A, raw data are in Table 1). On the contrary, childhood/young T1DM patients had significantly higher levels of cEPCs (13.90 ± 1.95 *n* = 59) compared to control subjects (4.92 ± 0.89 *n* = 11; *p* < 0.001, Mann–Whitney *U* test) (Figure 3B, raw data are in Table 2). In both cohorts, no statistically significant association emerged between the number of cEPCs and available clinical parameters, such as gender, percentage of glycated Hb, and body mass index (BMI).

**Table 1:** Clinical characteristics of adult type 1 diabetes mellitus (T1DM) patients and control subjects enrolled for the setting up of circulating endothelial progenitor cell (cEPC) detection and evaluation.

Patient ID	Gender	T1DM duration (years)	cEPC	Age	HbA1c (%)	BMI	Body weight (kg)	Height (cm)	Fat mass %	Lean mass %	CHO	HBGI	LBGI
A01	Female	13	6.1	45	7.0	19.03	55.0	170	22.2	77.8	Yes	11.5	1.5
A02	Female	9	5.4	38	8.7	28.08	91.0	180	20.8	79.2	Yes	10.8	1.4
A03	Female	4	6.3	38	8.1	23.14	63.0	165	27.7	72.3	Yes	10.6	1.3
A04	Male	20	4.0	47	7.2	27.40	96.0	190	24.0	76.0	Yes	3.8	2.8
A05	Female	33	8.4	33	7.2	18.80	53.0	168	18.1	81.9	Yes	11.1	1.6
A06	Male	26	8.1	43	7.2	21.90	67.0	175	19.2	80.8	Yes	7.2	4.1
A07	Female	26	3.2	59	7.3	20.60	61.0	172	26.4	73.6	Yes	4.15	5.17
A09	Female	5	4.2	37	8.4	22.60	53.0	153	27.3	72.7	Yes	11.3	1.6
A10	Female	6	3.3	31	7.4	21.10	55.4	162	26.4	73.6	Yes	8.6	6.5
A11	Female	23	2.8	35	7.4	27.10	65.0	155	26.7	73.3	Yes	6.2	3.8
A12	Female	21	3.9	36	6.8	20.40	61.0	173	26.3	73.7	Yes	4.3	4.2
B01	Male	2	5.8	22	7.0	22.78	72.0	178	11.4	88.6	No	10.1	6.5
B02	Male	32	1.2	59	7.5	20.22	53.0	162	17.4	82.6	No	12.2	1.9
B03	Female	28	0.6	40	8.5	25.17	70.0	167	30.5	69.5	No	10.8	1.4
B04	Female	2	4.4	28	6.8	21.09	52.0	157	19.2	80.8	No	11.3	1.6
B05	Male	2	5.6	27	6.5	24.44	74.0	174	26.4	73.6	No	10.8	1.5
B06	Female	2	4.8	25	7.0	18.53	48.0	161	11.5	88.5	No	3.8	2.8
B07	Male	10	4.4	24	7.5	27.04	85.0	188	23.1	76.9	No	12.1	1.8
B08	Female	22	5.1	37	7.0	21.30	61.0	170	19.4	80.6	No	6.2	3.0
B09	Female	2	5.3	43	10	20.70	55.0	163	20.1	79.9	No	10.6	1.3
B10	Male	11	6.0	39	6.5	25.4	75.0	172	26.3	73.7	No	10.3	1.2
B11	Female	6	0.8	42	8.0	22.3	60.0	164	23.3	76.7	No	11.6	1.5
B12	Female	22	2.2	39	6.4	22.00	65.0	172	26.4	73.6	No	7.6	4.5
C01	Male	Control	11.2	48									
C02	Female	Control	9.4	39									
C03	Male	Control	8.2	51									
C04	Male	Control	7.9	46									
C05	Male	Control	9.4	45									
C06	Female	Control	11.0	28									
C07	Male	Control	11.4	38									
C08	Male	Control	11.8	46									
C09	Male	Control	11.6	48									
C10	Female	Control	10.9	49									
C11	male	Control	10.4	37									

*BMI, body mass index; CHO, carbohydrate counting; HBGI, high blood glucose index; LGBI, low blood glucose index*.

**Table 2:** Clinical characteristics of childhood/young type 1 diabetes mellitus (T1DM) patients enrolled in the study.

Patient ID	Gender	T1DM duration (years)	cEPC	Age	HbA1c	BMI	Glycemia	I/W
PD 001	Male	14	9.2	19	6.5	21.10	67	0.79
PD 002	Male	4	32.6	17	6.6	ND	100	0.68
PD 003	Male	4	16.7	8	6.8	16.40	164	0.65
PD 004	Female	6	9.2	18	8.5	21.60	239	1.16
PD 005	Male	5	2.3	13	9.0	17.30	205	0.74
PD 010	Female	4	8.3	15	7.1	25.70	197	0.45
PD 012	Male	2	0.7	8	7.3	16.40	182	0.84
PD 013	Female	12	11.7	16	8.3	22.90	226	0.82
PD 020	Male	3	0.3	7	9.7	ND	250	ND
PD 021	Male	9	3.0	19	9.8	24.40	90	0.75
PD 022	Male	I4	43.3	19	8.6	24.40	271	0.62
PD 025	Female	10	3.6	14	7.8	21.40	142	1.00
PD 032	Male	5	4.5	7	7.5	14.50	119	0.70
PD 033	Female	1	1.8	15	6.3	25.10	126	0.72
PD 035	Male	2	40.3	17	5.7	23.60	186	0.40
PD 037	Female	4	6.8	14	8.2	17.70	212	0.90
PD 040	Male	2	2.7	12	7.6	20.70	107	0.93
PD 041	Male	2	11.9	8	7.5	15.80	114	0.67
PD 042	Female	14	23.4	17	7.5	18.00	289	1.03
PD 044	Female	2	75.0	16	6.5	24.80	108	0.88
PD 045	Male	2	10.2	9	6.5	16.90	122	0.76
PD 046	Male	5	18.3	18	6.7	19.40	179	0.83
PD 047	Male	8	4.5	18	7.2	23.20	164	0.70
PD 048	Female	2	7.6	16	6.0	20.90	117	1.14
PD 049	Female	1	14.0	9	8.0	23.40	208	1.28
PD 050	Female	5	10.6	14	8.0	29.90	102	0.92
PD 051	Female	9	16.4	13	7.4	18.00	304	1.05
PD 054	Male	10	45.8	15	9.5	24.10	220	1.08
PD 055	Female	4	17.5	17	7.7	ND	365	ND
PD 056	Female	5	17.9	10	8.1	15.60	368	0.74
PD 058	Male	4	20.2	15	10.3	ND	270	0.82
PD 063	Female	4	38.3	17	7.5	26.90	164	0.84
PD 064	Male	11	8.4	14	8.1	17.80	83	0.71
PD 065	Female	9	19.0	10	7.5	14.40	174	0.92
PD 066	Male	4	24.2	11	8.0	18.10	205	0.92
PD 067	Male	8	38.6	11	7.3	17.40	102	1.02
PD 068	Female	2	30.4	10	8.7	19.70	203	1.08
PD 069	Female	4	11.3	14	7.2	21.60	270	0.98
PD 070	Female	12	34.4	19	8.7	27.10	163	0.61
PD 071	Male	1	26.8	10	6.8	17.20	146	0.89
PD 073	Male	11	2.4	18	7.3	22.80	183	0.73
PD 074	Male	12	0.3	17	7.0	29.30	106	0.86
PD 075	Male	4	3.3	18	6.1	ND	155	ND
PD 080	Male	5	1.4	16	10.2	19.90	333	1.16
PD 081	Male	8	16.5	14	7.0	30.40	319	0.90
PD 083	Female	9	8.2	13	7.8	18.20	334	1.00
PD 084	Female	2	12.5	17	6.4	22.30	104	0.69
PD 085	Female	1	25.7	14	8.0	23.30	181	0.54
PD 086	Male	6	35.0	10	8.0	15.60	165	0.55
PD 087	Female	1	2.9	6	6.8	15.40	223	0.07
PD 088	Male	10	7.1	13	8.0	17.20	210	1.06
PD 090	Male	8	23.4	15	7.8	19.20	178	0.93
PD 091	Male	1	2.6	16	8.0	21.60	243	0.40
PD 093	Female	2	3.7	10	7.6	17.50	173	0.82
PD 094	Male	4	6.2	16	9.0	23.80	279	0.77
PD 097	Female	2	3.5	16	7.7	26.10	185	0.58
PD 099	Male	13	42.0	19	7.9	ND	54	ND
PD 101	Female	10	4.2	14	7.0	21.50	71	1.06
PD 112	Male	2	5.3	10	7.2	19.10	243	0.74
PD 006	Male	Control	18	14				
PD 007	Female	Control	3.1	15				
PD 008	Male	Control	4.0	11				
PD 017	Female	Control	2.3	15				
PD 018	Male	Control	1.5	7				
PD 019	Male	Control	3.6	8				
PD 057	Male	Control	8.7	3				
PD 103	Male	Control	6.2	7				
PD 108	Female	Control	0.2	11				
PD 113	Male	Control	1.7	18				
PD 114	Male	Control	4.8	7				

*BMI, body mass index; I/W, insulin/weight*.

**Figure 4:**
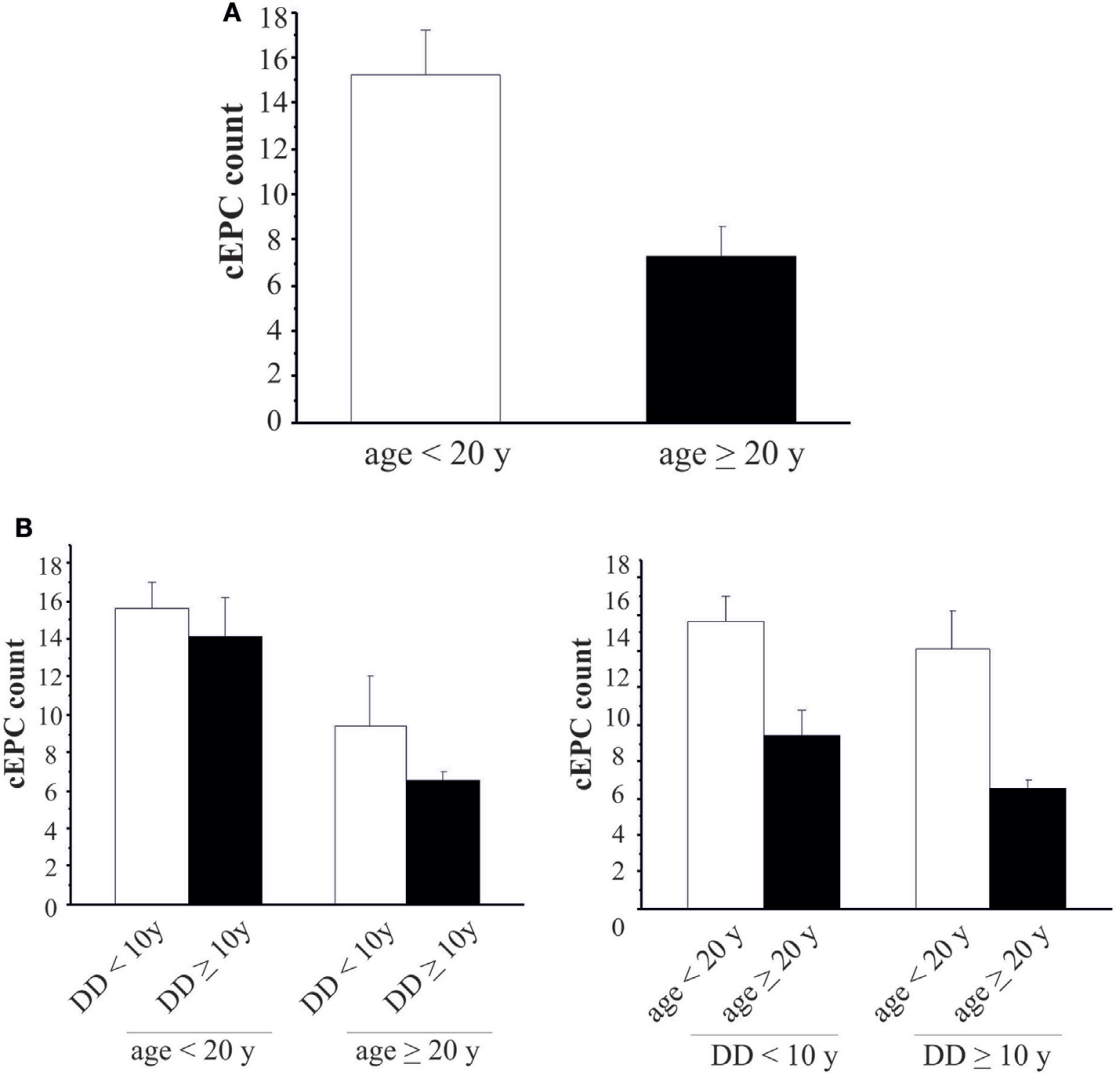
**(A)** Circulating endothelial progenitor cell (cEPC) counts in type 1 diabetes mellitus patients of different age groups (white bar: age <20 years; black bar: age ≥20 years). Data are reported as mean ± SEM; *p* = 0.004. **(B)** Histograms summarizing cEPC levels in patients belonging to the different age and disease duration groups. Data are reported as mean ± SEM. Left histogram: *p* = 0.689 and *p* = 0.418, Mann–Whitney *U* test. Right histogram: *p* = 0.153 and *p* = 0.061, Mann–Whitney *U* test.

We then enrolled 28 T1DM patients from either centers (Table 3), so that the study population was: 52 patients in the ≥20 years group and 59 in the <20 years group. In order to evaluate whether the number of cEPCs was anyhow related to the patients’ age and/or with the duration of the disease T1DM patients from both centers were grouped into two age categories: patients younger (<20 years) or older than 20 years (≥20 years). In agreement with data shown in Figure 3, the number of cEPCs turned out to be significantly higher in T1DM patients younger than 20 years with respect to older patients (15.24 ± 1.12 *n* = 59 vs 7.27 ± 0.73 *n* = 52; *p* = 0.004, Mann–Whitney *U* test) (Figure 4A).

**Table 3:** Clinical characteristics of adult and childhood/young type 1 diabetes mellitus (T1DM) patients enrolled to complete the cohort under study.

Center	Patient ID	T1DM duration (years)	cEPC	Age
*PO*	AD 001	14	7.5	49
*PO*	AD 002	12	5.6	50
*PO*	AD 003	14	9.7	40
*PO*	AD 004	12	3.1	47
*PO*	AD 005	18	11.2	35
*PO*	AD 006	11	4.3	43
*PO*	AD 007	10	1.4	32
*PO*	AD 008	13	3.4	38
*PO*	AD 009	20	3.6	38
*PO*	AD 010	18	4.8	32
*PO*	AD 011	22	9.3	56
*PO*	AD 012	28	5.8	39
*PO*	AD 013	30	8.4	42
*PO*	AD 015	32	6.6	42
*AOUM*	PD 011	5	1.2	24
*AOUM*	PD 023	18	4.1	21
*AOUM*	PD 029	6	66.2	22
*AOUM*	PD 031	12	12.5	20
*AOUM*	PD 036	14	10.4	27
*AOUM*	PD 038	13	22.3	23
*AOUM*	PD 052	12	11.1	21
*AOUM*	PD 059	12	15.0	20
*AOUM*	PD 060	12	13.1	22
*AOUM*	PD 092	12	1.2	25
*AOUM*	PD 096	9	10.1	22
*AOUM*	PD 100	11	8.0	21
*AOUM*	PD 109	39	1.5	42
*AOUM*	PD 111	11	0.6	20

**Figure 5:**
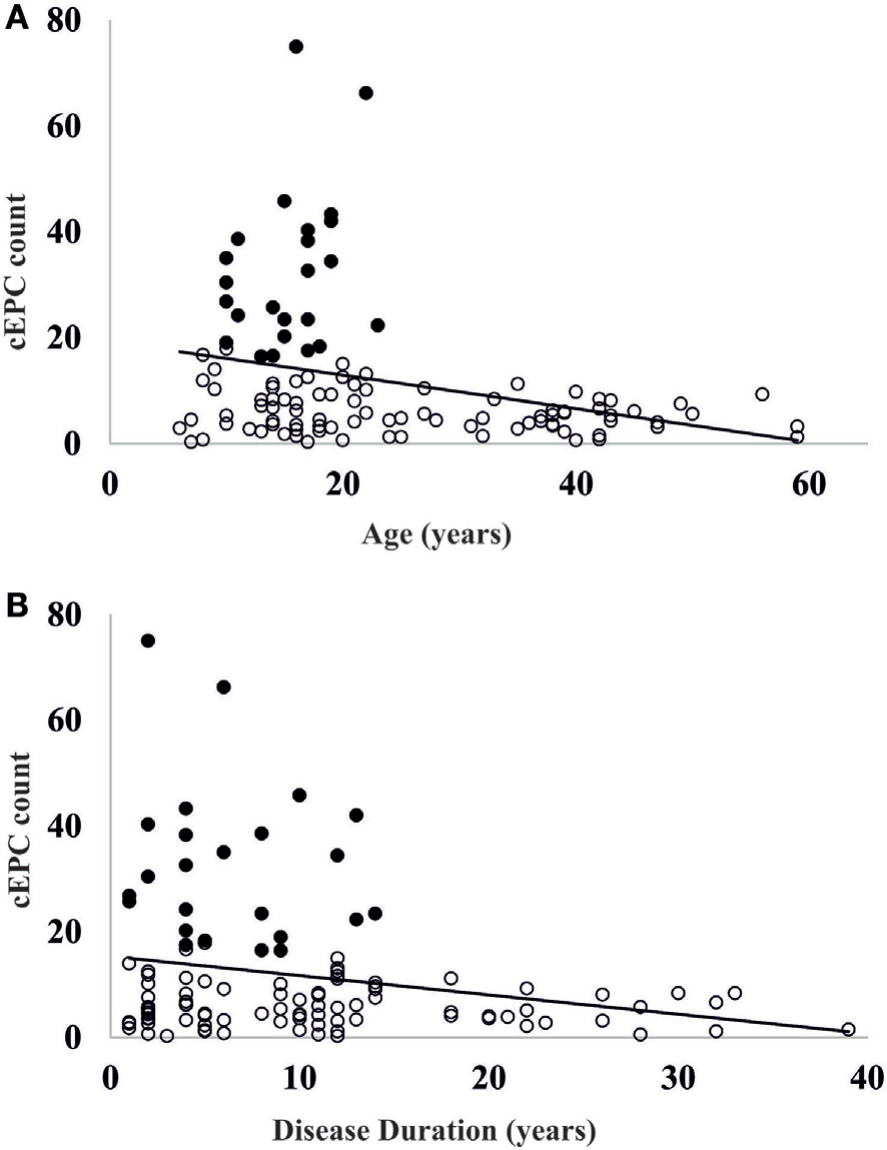
**(A)** Scatter plot of the distribution of circulating endothelial progenitor cell (cEPC) vs age. Black circles: patients with high endothelial progenitor cell (EPC) count. **(B)** Scatter plot of cEPC vs disease duration. Black circles: patients with high EPC count.

Once demonstrated that the number of circulating EPCs falls with the increasing of age, we evaluated whether the differences in cEPCs levels depended only on the age of the patient or also on the duration of the disease. To this purpose, we categorized T1DM patients into the two groups depending either on the age (≥ or <20 years) or on the duration of the disease (shorter or longer than 10 years, DD < 10 years or DD ≥ 10 years) and divided each group into two subgroups, based on DD and age, respectively. No differences were found between DD subgroups when the patients were categorized by age (Figure 4B, left panel; individual means and *p* values are in figure legend). When the patients were categorized on the basis of disease duration, the difference in cEPC counts between <20 and ≥20 years patients was roughly of the same entity in both DD groups, although with a lower *p* value in the DD ≥ 10 years group (Figure 4B, right panel; individual means and *p* values are in figure legend). Furthermore, we plotted the individual cEPC count values vs either patients’ age or duration (Figure 5). A subset of childhood/young patients, aged less than 20 years (and with a disease duration less than 10 years) emerged with very high cEPC counts (black shaded circles in Figure 5).

## Discussion

In this study, we determined the number of cEPCs in T1DM patients without clinical vascular damage, of different ages and disease duration. In the first step of the study, cEPCs were measured in two separate T1DM patients’ groups, either adults or pediatric, in comparison with controls of the same age range. In the second step, patients were grouped in two age groups (≥ or <20 years) and the number of cEPCs was correlated with both the age and the duration of the disease. We provide evidence that in T1DM patients without cardiovascular complications, the number of cEPCs is significantly correlated with patients’ age, whereas does not depend on other clinical parameters, such as metabolic control (HbA1c), glycemic variability (MAGE), and BMI.

The number of cEPCs in adult T1DM patients turned out to be lower compared to both age-matched controls and to childhood/young T1DM patients. The latter showed very high levels of cEPCs compared to age-matched controls. The lower amount of cEPCs found in adult T1DM patients agrees with previous studies in both T1DM (20–23) and T2DM patients (9, 11–13), while the high number of cEPCs in pediatric T1DM patients is in line with what reported by Głowińska-Olszewska et al. (25) in T1DM children. Hence, the apparent contradicting data in cEPC number reported in T1DM patients in the literature could be reconciled considering the age of the patients. The number of cEPCs apparently changed also in normal subjects. In fact, although in a small number of control subjects, we found an increase in the number of cEPCs from children to adults. Most of the data in the literature show a decrease in cEPC number in aged subjects (27, 28) and correlate this decrease with impairment in endothelial repair and onset of vascular damage (29). Our data, along with current literature, suggest the necessity to consider an age-related profile of cEPC production in physiological conditions. The progressive increase in cEPC number until young/adult age, and their progressive decrease in the elderly, could also determine differences in age-dependent cEPC production in pathological conditions.

Another aspect that must be considered when studying the number of cEPCs in different conditions is represented by the different methodologies used to analyze and quantify them. In fact, the phenotypical marker of EPCs is CD133 that is absent on mature endothelial cells, and other surface markers are CD34^+^, VEGFR2^+^, and CD146^+^ (30). However, to date, there are no standardized methods to quantify and identify EPCs and the protocols used vary between studies (31). Moreover, most studies in both T2DM and T1DM patients were based on the determination of EPCs based on their growth *in vitro* (20).

Finally, the different kinds of therapies with insulin (dose and number of administration and duration of therapy) could also contribute to the different cEPC profiles in T1DM, as shown for T2DM patients (32). However, we did not find any association between the number of cEPCs and the insulin to weight ratio in the pediatric cohort analyzed in the present study.

Besides differences in cEPC number compared to controls, the most interesting result of the present study is that childhood/young (<20 years) T1DM patients have a higher number of cEPCs compared to adult (>20 years) patients. Such higher number of cEPCs is maintained also when the disease duration is longer than 10 years (Figure 4B, right panel). Moreover, a subpopulation of childhood/young T1DM patients, aged <20 years, whose disease lasts for less than 10 years are characterized by very high cEPC values. It is tempting to speculate whether these young patients with high cEPC levels could be protected against endothelial dysfunction. On the contrary, when the disease occurs in older age, the low levels of cEPCs could mirror a lowering of such protective effect. These still preliminary data would support the clinical observation of less incidence of late vascular complications in T1DM when the onset of the disease is in childhood respect to adult age. We wonder whether these effects could be related to a better glycemic control obtained in pediatric patients. In Hörtenhuber’s prospective study, an increase in cEPC number after one year was reported in association with better glycemic control (24). In line with these results, Marfella et al. (17) showed that during percutaneous coronary intervention, an optimal peri-procedural glycemia control improves myocardial salvage, by increasing cEPC number and their capability to differentiate. Notably, the same group (16) had shown that a poor glycemic control reduces EPC number in T2DM, through a mechanism that is mediated by Sirtuin expression (19).

## Conclusion

The present study shows that a relevant association exists between the number of cEPCs and the age and duration of the disease in T1DM patients. One of the limitations of the present study is the relatively small number of patients and short follow up, as well as the lack of data on cEPC functionality, that render the data still preliminary. Nevertheless, if appropriately circumstantiated in a further study, our results might provide an additional explanation to the pathogenesis of complications in T1DM as well as to the clinical evidence of less complications in T1DM patients when the onset of the disease is in the pediatric age. Moreover, in agreement with current literature, our data suggest that maintaining a high number of cEPCs, possibly through a good glycemic control, would contribute to contain the CVD burden in T1DM.

## Ethics Statement

The study was approved by Local Ethical Committee.

## Author Contributions

AdA, AA, and ST conceived and designed the work. MD, EL, SP, BP, LL, MC, AdA, AA, and ST acquisited, analyzed, and interpreted the data for the work. AdA, AA, and EL drafted the work or revised it critically for important intellectual content. AdA, AA, ST, EL, MD, SP, MC, BP, and LL gave final approval of the version to be published.

## Conflict of Interest Statement

The authors declare that the research was conducted in the absence of any commercial or financial relationships that could be construed as a potential conflict of interest.
